# Connecting dots in disorders of gut-brain interaction: the interplay of stress and sex hormones in shaping visceral pain

**DOI:** 10.3389/fpsyt.2023.1204136

**Published:** 2023-05-19

**Authors:** Franziska Labrenz, Christian J. Merz, Adriane Icenhour

**Affiliations:** ^1^Department of Medical Psychology and Medical Sociology, Ruhr University Bochum, Bochum, Germany; ^2^Department of Cognitive Psychology, Institute of Cognitive Neuroscience, Ruhr University Bochum, Bochum, Germany

**Keywords:** cortisol, gonadal hormones, gut-brain axis, pain, sex differences, visceroception

## Abstract

Visceral pain and stress are tightly intertwined bodily and emotional phenomena, which enable a flexible adaptation to environmental challenges by activating a response repertoire to restore homeostasis along the gut-brain axis. However, visceral pain and stress can persist widely independent of the initial cause, acquiring independent disease values and posing major health burdens as predominant features in disorders of gut-brain interaction (DGBI). Epidemiological data consistently documents an increased prevalence for women to suffer from chronic visceral pain, possibly shaped by sex hormones and modulated by stress and its biological and psychosocial correlates. Yet, mechanisms underlying the complex interactions between altered visceroception, stress and sex remain widely elusive, especially in clinical populations with DGBI.

We herein selectively review mechanisms of interactions between stress and sex in the complex pathophysiology of DGBI. A particular emphasis is laid on visceral pain, in which stress constitutes a major risk factor as well as mediator, and sex-related differences are particularly pronounced. Building on the neurobiology of stress and mechanisms of gut-brain interactions, we highlight putative target mechanisms *via* which visceral pain and stress may converge with sex effects into a triad.

Accommodating a global demographic shift, we propose a lifespan perspective in future research, which may enable a more fine-tuned evaluation of this complex interplay exerting distinct challenges during vulnerable developmental phases. This viewpoint may advance our understanding of pathophysiological processes and can ultimately inspire novel tailored prevention strategies and therapeutic approaches in the treatment of chronic visceral pain and DGBI across the lifespan.

## 1. Introduction

Pain and stress are closely interwoven phenomena with shared conceptual underpinnings, psychological mechanisms and physiological responses (1). Their mutual influences on biological and psychosocial levels enable a flexible adaptation to environmental challenges by activating a response repertoire aimed to restore homeostasis and to regulate health (2, 3). However, both pain and stress can persist widely independent of the initial cause, losing their protective function, and acquiring an independent disease value, which substantially compromises quality of life.

In the context of DGBI, most evidence on the key role of stress and its neurobiological correlates comes from research on irritable bowel syndrome (IBS). With prevalence rates of up to 10% worldwide (4), IBS is not only the most common chronic visceral pain disorder but also exemplary of disturbed gut-brain interactions (5). Constituting key factors in the etiology and pathophysiology of the disease, stress and visceral pain often co-occur in IBS and can exert reciprocal effects. On the one hand, acute stress can increase pain sensitivity in patients (6, 7), affect gut motility and IBS symptomatology [reviewed in (8)] and alter patients’ neural responses to experimental visceral pain (9). On the other hand, pain *per se* constitutes a meaningful stressor able to trigger systemic changes in neural, neuroendocrine, and immunological systems, which in turn can profoundly impact on gut-brain communication (10). Particularly if stress persists, these systems can become dysregulated, resulting in exaggerated allostatic load (11) and altered stress reactivity (12). As such, stress and its neurobiological correlates demonstrably act as both, relevant risk factors for disease development (13–16) as well as accelerators of symptoms and symptom burden (7, 17, 18), deeming IBS a stress-related condition.

Importantly, women seem to be more prone than men to the development of conditions characterized by pain and stress alike (19, 20). These observations support sex-related factors to play major roles in the transition from adaptive to maladaptive responses and at the same time suggest complex interactions between pain, stress and sex in pathology. Sex differences are most pronounced in the context of visceral pain in disturbances of the gut-brain axis (21, 22) in which, interestingly, stress and its direct and indirect effects on central and peripheral pathways in gut-brain communication constitute key players (23).

While epidemiological data are widely consistent, experimental studies targeting the mechanisms underlying interactions between pain, stress and sex mainly in young healthy participants reveal enormous inter-individual variability and do not allow the clear conclusion that women are *per se* more sensitive to either stress (24) or pain (25, 26), including visceral pain (27). Especially in the context of complex gut-brain interactions, these inconsistencies underscore the need to systematically identify and target modulating biological and psychosocial factors to gain further insights into the triad of visceral pain, stress and sex and its impact on DGBI.

Based on the neurobiology of stress and its role within the gut-brain axis, we herein selectively review current knowledge regarding sex effects on the interaction between visceral pain and stress and highlight putative underlying mechanisms. Accommodating a demographic shift observed worldwide, we particularly propose a lifespan perspective in future experimental and clinical research to advance our understanding about sexually dimorphic effects in DGBI and stress-related disorders, which may ultimately inspire tailored prevention and resilience strategies particularly during vulnerable developmental phases.

## 2. The gut-brain axis

**Figure 1:**
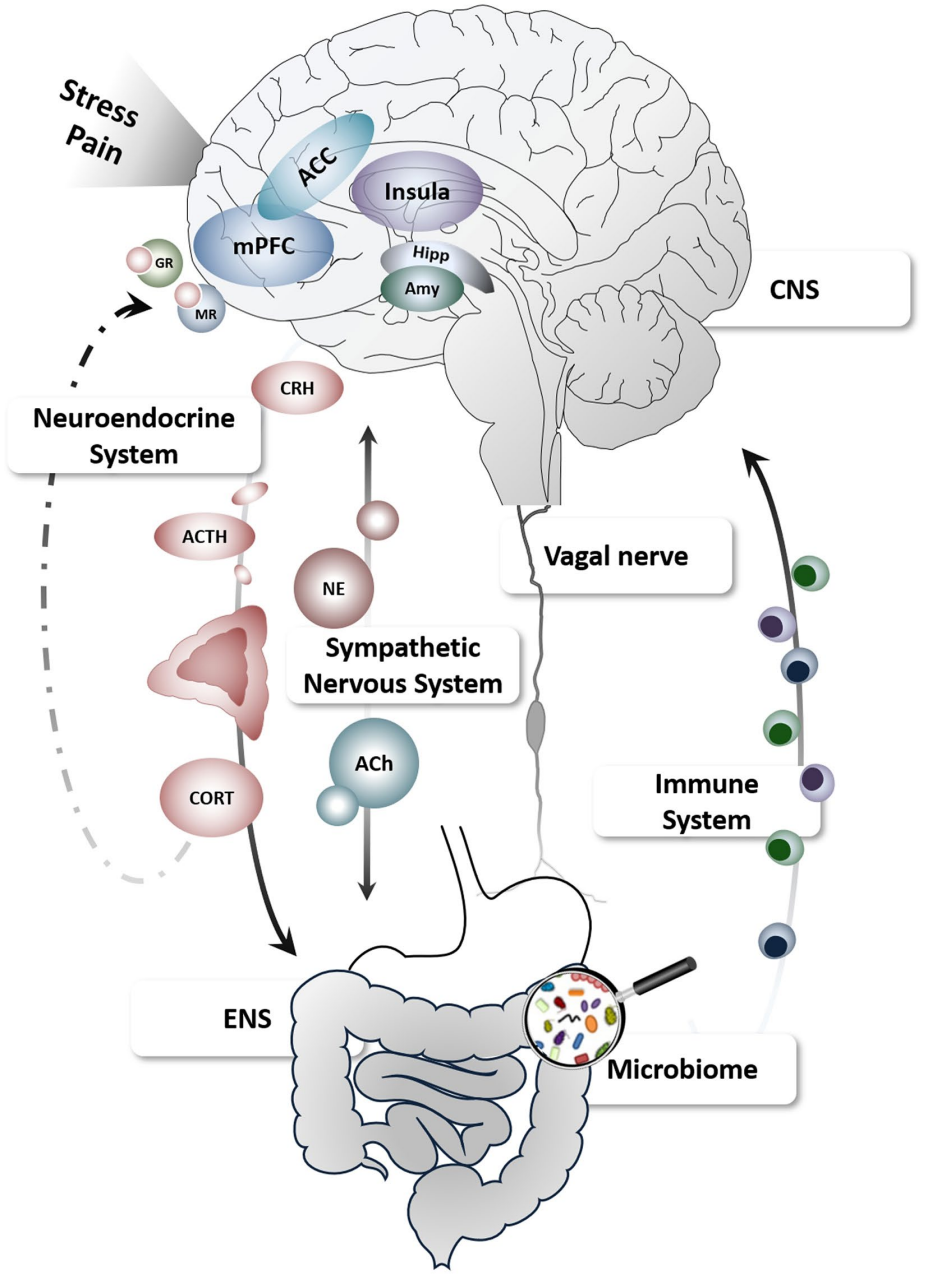
Schematic depiction of the gut-brain axis and key pathways relevant to stress and pain. Figure created using motifolio templates (www.motifolio.com). ACC, anterior cingulate cortex; ACh, acetylcholine; ACTH, adrenocorticotropic hormone; Amy, amygdala; CNS, central nervous system; CORT, cortisol; CRH, corticotropin-releasing hormone; ENS, enteric nervous system; GR, glucocorticoid receptors; Hipp, hippocampus; mPFC, medial prefrontal cortex; MR, mineralocorticoid receptors; NE, norepinephrine.

The gut-brain axis (Figure 1) conceptualizes the complex and multidirectional communication pathways connecting the brain and gastrointestinal tract, involving modulators within a bio-psychosocial framework (28). Growing evidence supports continuous crosstalk between the enteric (ENS) and central nervous system (CNS) along vagal, spinal (afferent) and humoral (efferent) pathways serving key functions in monitoring and maintaining homeostasis, detecting challenges to the organism and restoring bodily integrity (29). As such, both ENS- and CNS-derived processes and their interactions along the gut-brain axis are capable to shape visceral symptoms including pain but can also modulate stress and stress effects.

The ENS is mainly under control of intrinsic enteric neurons and glia, smooth muscles and the lamina propria of the mucosa, but is also extrinsically innervated by primary afferent and autonomic fibers connecting the gastrointestinal tract with the spine and brain (30, 31). The brain together with neuroendocrine, immune, and autonomic nervous systems constitute profound modulators of motility and gut function outside the ENS. In addition, multiple psychological mechanisms including cognitive and emotional factors are integrated with ascending sensory information in distinct brain circuits involving the insula and anterior cingulate cortex as core nodes of the salience network (32), which ultimately shape subjective experiences, as well as autonomic and behavioral responses to visceral sensations (33, 34). Conversely, ENS mechanisms can themselves exert powerful effects on these pathways and brain functions, providing several afferent and efferent target routes for stress-induced modulations of visceral symptoms (35). Finally, increasing evidence supports a key role of gut microbial composition not only in intestinal barrier functioning but also in brain function and behavior. Along this gut-brain-microbiota axis, stress appears to be a prominent modulator *via* immunological, endocrine, and neural communication channels, deeming stress a key player in this emerging field in the neurosciences (36).

Effects of stress on gastrointestinal function observed in preclinical and clinical studies in patients suffering from chronic visceral pain demonstrably involve alterations along several gut-brain pathways (23, 37). Mediators of the stress response system have been linked to stress-induced changes in intestinal permeability (38), gut motility and visceral hyperalgesia (39), possibly involving inflammatory mechanisms such as mast cell activation and release of pro-inflammatory cytokines (40). Using well-established experimental stress models such as public speech or dichotomous listening, few studies in healthy humans demonstrated acute increases in gut permeability (41, 42). Furthermore, increasing evidence using various experimental stressors supports the notion that stress can induce profound changes in emotion, mood and cognition (14, 16, 43), including effects on brain function (44).

Importantly, applying an experimental visceral pain model with rectal distensions in healthy volunteers, stress and stress mediators were also found to alter visceral perception, particularly aversive visceral symptoms including pain (14, 16) with distinct effects of sex as well as the type of stress model. Specifically, young healthy men and women did not seem to *per se* differ with respect to visceral sensitivity (27) and sensitivity to aversive visceral sensations was equally lowered in individuals being subject to increased chronic stress load (14). However, pharmacologically increased cortisol concentrations resulted in lowered visceral (but not somatic) pain thresholds particularly in women (16). While warranting further research to substantiate these findings, these first data indicate that sex and sex hormones may directly interact with neurobiological stress modulators in distinctly shaping visceral perception along the gut-brain axis in both health and DGBI (45).

## 3. The neurobiology of stress

In general, stress sets a fine-tuned orchestration of affective, physiological, immunological and endocrine responses into motion helping the organism to adequately respond to or prepare for a (potential) stressor. The acute stress response is adaptive, while chronic stress represents a potent risk factor for developing mental and somatic disorders (46, 47), including chronic pain (48). Two major systems govern the stress response: first, activation of the sympathetic nervous system leads to a rapid release of (nor)epinephrine from the adrenal medulla and sympathetic nerves (11) increasing heart rate and blood pressure. Second, stress activates the hypothalamus-pituitary-adrenocortical (HPA) axis with an initial release of corticotropin-releasing hormone (CRH) from the hypothalamus. In the anterior pituitary, CRH stimulates the secretion of adrenocorticotropic hormone into the bloodstream, which leads to the release of glucocorticoids, in humans mainly cortisol, from the adrenal cortex. Glucocorticoids pass the blood-brain barrier and occupy mineralocorticoid and glucocorticoid receptors in a plethora of brain regions such as the amygdala, hippocampus or prefrontal cortex (49–52).

Importantly, stress responses differ between men and women (53, 54) and depend on sex hormone availability. For example, reduced cortisol release was observed during the follicular phase of the menstrual cycle, characterized by low female sex hormone concentrations, in comparison to the luteal phase with high concentrations of estradiol and progesterone (55). Intake of hormonal contraceptives leading to low endogenous sex hormone availability also reduces or even blunts cortisol release to acute stress (56), which is, however, restricted to the free, biologically active part of cortisol measured in saliva. Interactions between stress and female sex hormones, including hormonal contraception, have been observed in a variety of processes such as episodic memory, fear conditioning and emotion regulation (57–59), which are also of relevance in the context of pain and its modulation (60–62). In addition to female sex hormones, androgens, particularly testosterone, also impact HPA axis activity, partially mediated by a conversion to estradiol (63). Evidence mainly obtained from rodent models suggests a protective role of testosterone in the context of pain (64–66), including visceral pain (67). However, the dearth of research in humans and partly inconsistent findings (68, 69) hinder clear conclusions regarding a distinct impact of testosterone and its interaction with stress in affecting visceral pain and DGBI. Together, while a putative reciprocity between pain, stress and sex is widely discussed as key in the tremendous interindividual variability in pain- and stress-related disorders (70–73), including DGBI (10, 74), the exact processes of interaction in this triad and underlying mechanisms remain widely elusive.

## 4. Sex, stress and pain in disorders of gut-brain communication—promoting a lifespan perspective

**Figure 2:**
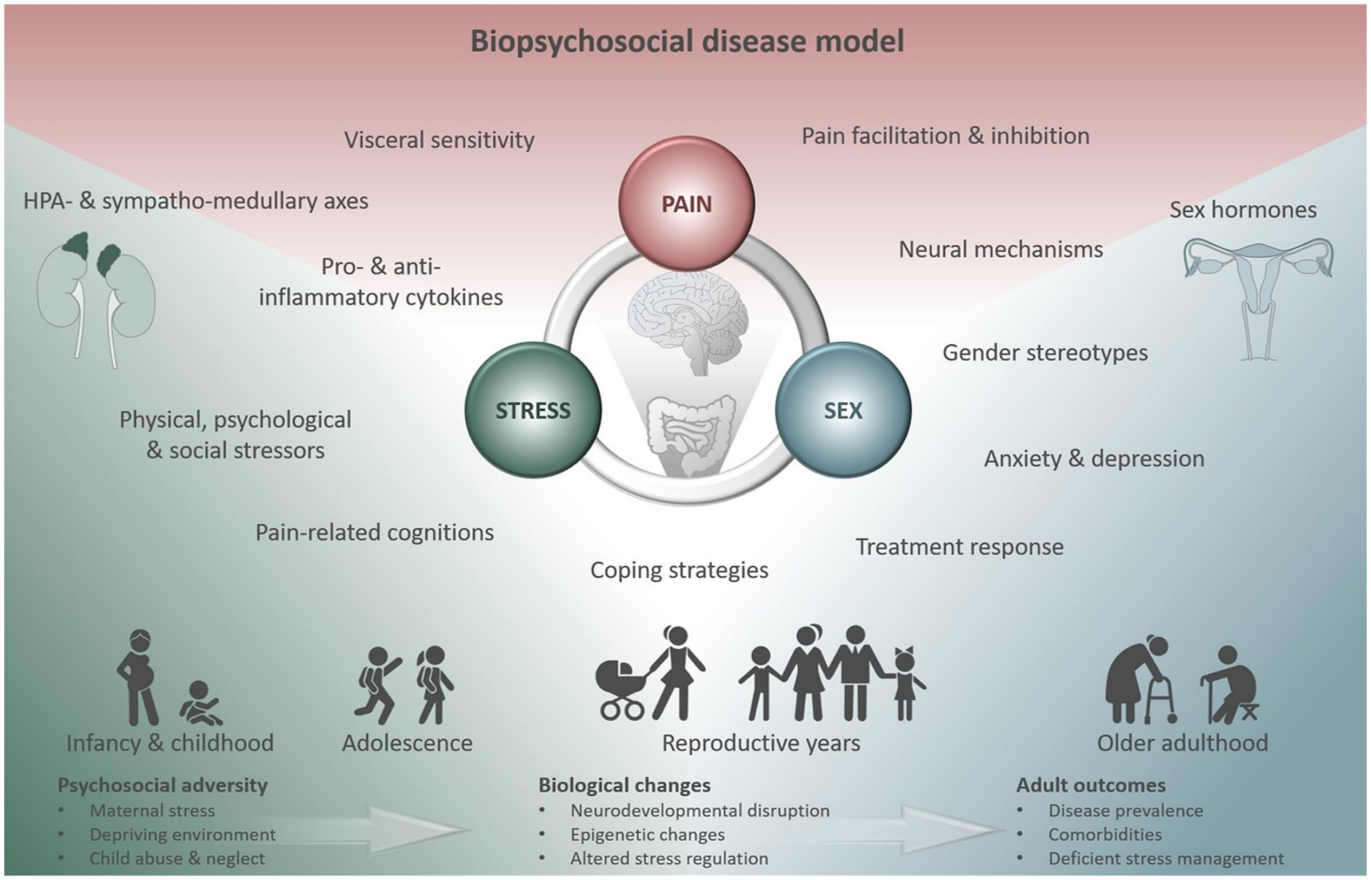
Biopsychosocial disease model summarizing multidimensional factors modulating the triad of visceral pain, stress and sex. Their interactions exert distinct effects in different vulnerable phases across the lifespan affecting prevalence, disease-related biological changes and comorbidities in adulthood. Figure created using motifolio templates (www.motifolio.com).

Particularly in the context of DGBI, both stress and female sex are consistently identified as relevant risk factors (10, 22, 75) and sex differences have been documented for prevalence, incidence, pathophysiologic factors, clinical characteristics, and response to therapy (76). Using a multidimensional approach, a supersystems perspective to describe the connection between pain and stress through reciprocal neural, endocrine, and immune interactions (77) has recently been extended to integrate dysregulations underlying sexually dimorphic effects into a biopsychosocial model (78) (Figure 2). Embracing this concept, distinct central nervous system processing, genetic factors, responsivity of the HPA and sympatho-medullary axes, as well as sex hormones and their influences across the menstrual cycle are undoubtedly involved in visceral pain and DGBI. However, their interplay with psychosocial aspects ranging from proneness to anxiety and depression and sexually dimorphic pain coping strategies to stereotypic gender roles along with cultural and environmental factors is likely what ultimately increases the vulnerability to chronic visceral pain in women (28, 75, 78). In other words, biopsychosocial influences may function as catalyzers to the extent in which stress impacts the development and persistence of pain symptoms along the gut-brain axis in a sexually dimorphic manner. Together, considerable efforts to account for the complexity of relations between visceral pain, stress and sex (hormones) have been made. However, a crucial factor gaining increasing attention in elaborate animal models, yet thus far widely neglected in both conceptual and empirical work in humans, are changes across the lifespan (79). Age-related effects may impact the interaction between visceral pain, stress and sex-related effects in multiple ways: Directly *via* changes in sex hormone concentrations or HPA axis activity across the lifespan (80, 81), indirectly by inducing secondary changes caused by, e.g., environmental and lifestyle factors or in moderating the extent of biopsychosocial factors affecting the individual, particularly during vulnerable periods of life (79).

For example, psychosocial adversity occurring pre- or postnatally, particularly when stress leads to significant or sustained responses of the stress and immune systems (82), can exert massive detrimental effects on the offspring, which may not unfold instantly, but can manifest during sensitive periods later in life. These manifestations appear to be sexually dimorphic with females tending to show increased passive coping, symptoms of anxiety and depression along with aberrant HPA responsivity (79), known to be key risk factors for the development of stress-related disorders including DGBI (83). Consistently, the experience of early adverse life events is able to reliably predict IBS status in women, particularly when associated with intense fear (13) and can have long-term consequences on brain structure and function (84), involving emotion regulation and salience networks in a sex-dependent manner in both health (85) and DGBI (86). Puberty, characterized by physiological changes related to sexual maturation and regulated by several endocrine and genetic factors, appears to be a second critical window when considering interactions between stress, sex and mechanisms along the gut-brain axis. Mainly hormonally driven, puberty is known to interact with the gut’s microbiome and sex-related differences in the microbial composition emerge at the onset of this sensitive developmental phase (87, 88). Given that stress can demonstrably interfere with the gut microbiota (89) and contribute to gut dysbiosis in a sex-specific manner (90), it appears plausible that stressors encountered during puberty may be critical with respect to the vulnerability of dysregulated gut-brain communication. Finally, while rapid changes in estrogen and progesterone levels such as across the menstrual cycle appear to be associated with an exacerbation of bowel symptoms (91), a long-term decline in ovarian hormone concentrations following menopause seems to be related to a decrease in the incidence of DGBI in women (92). This effect, resulting in the elimination of prevalence differences of visceral pain in middle to older aged men and women (22), may, however, be reversed in those women under hormone replacement therapy (93), further supporting the key role of female sex hormones and their fluctuation in the vulnerability to chronic visceral pain conditions.

Together, while challenging, increasingly well-established age-related effects in all dimensions forming the triad of sex (94), stress (95) and visceral pain (96) call for future research particularly considering phenomena related to the lifespan when investigating and evaluating complex sex-stress-pain interactions in healthy humans and in patients suffering from DGBI.

## 5. Challenges and future directions

Tremendous inter individual variability in experimental and particularly clinical visceral pain in DGBI poses an enormous challenge in health care and effective treatment (36, 97). A biopsychosocial disease model is best suited to conceptualize the complex mosaic of individual and combined influences in moderating and mediating pain experiences and risk for chronification (70). Within this framework, experimental work and translational approaches should ideally be designed to simultaneously assess central, neuroendocrine, immunological and enteric mechanisms. Particularly, effects of acute and chronic stress burden, stress hormones and sex effects in terms of hormonal influences as well as psychosocial factors and the abovementioned interactions need be considered for a holistic appreciation of complex chronic pain conditions. Embedded within these approaches, embracing a lifespan perspective in terms of long-term follow-ups, longitudinal investigations and a particular emphasis on periods in life, which are especially vulnerable to stress-induced insults or associated with dynamic changes in hormonal levels is likely a promising future endeavor.

At first sight, age and changes across the lifespan appear to add yet another level of complexity onto the multifactorial etiology and pathophysiology of disturbed gut-brain interactions, in which several mechanisms are far from understood and others are likely still awaiting their discovery. However, in light of challenges arising from demographic changes, vast interest in mechanisms related to healthy aging is continuously growing (98). Ultimately, joint forces in transdisciplinary research to connect the dots between visceral pain, stress and sex from a lifespan perspective are therefore crucial to inspire transdisciplinary research, identify individualized therapeutic targets, and provide refined approaches for personalized prevention and treatment.

## Author contributions

AI and CJM acquired funding. FL, CJM and AI drafted the manuscript. All authors contributed to the article and approved the submitted version.

## Funding

This work was funded by the German Research Foundation (Deutsche Forschungsgemeinschaft, DFG), SFB 1280 *Extinction Learning* (316803389—projects A09 and A10).

## Conflict of interest

The authors declare that the research was conducted in the absence of any commercial or financial relationships that could be construed as a potential conflict of interest.

## Publisher’s note

All claims expressed in this article are solely those of the authors and do not necessarily represent those of their affiliated organizations, or those of the publisher, the editors and the reviewers. Any product that may be evaluated in this article, or claim that may be made by its manufacturer, is not guaranteed or endorsed by the publisher.
